# Integrated Chassis Control of Active Front Steering and Yaw Stability Control Based on Improved Inverse Nyquist Array Method

**DOI:** 10.1155/2014/919847

**Published:** 2014-03-20

**Authors:** Bing Zhu, Yizhou Chen, Jian Zhao

**Affiliations:** ^1^State Key Laboratory of Automotive Simulation and Control, Jilin University, Changchun 130022, China; ^2^Key Laboratory of Bionic Engineering of Ministry of Education, Jilin University, Changchun 130022, China

## Abstract

An integrated chassis control (ICC) system with active front steering (AFS) and yaw stability control (YSC) is introduced in this paper. The proposed ICC algorithm uses the improved Inverse Nyquist Array (INA) method based on a 2-degree-of-freedom (DOF) planar vehicle reference model to decouple the plant dynamics under different frequency bands, and the change of velocity and cornering stiffness were considered to calculate the analytical solution in the precompensator design so that the INA based algorithm runs well and fast on the nonlinear vehicle system. The stability of the system is guaranteed by dynamic compensator together with a proposed PI feedback controller. After the response analysis of the system on frequency domain and time domain, simulations under step steering maneuver were carried out using a 2-DOF vehicle model and a 14-DOF vehicle model by Matlab/Simulink. The results show that the system is decoupled and the vehicle handling and stability performance are significantly improved by the proposed method.

## 1. Introduction

Vehicle safety and stability has been one of the hottest research topics during last several decades. Many active control systems such as antilock brake system (ABS), traction control system (TCS), electric stability control (ESC), and active front steering (AFS) were developed and widely equipped on various vehicles for safer, more stable, and comfortable driving experience. However, as the complexity of vehicle active control systems increases, the potential conflicts among each system become increasingly problems and concerns [1–3].

The primary objective of the chassis control systems is to improve vehicle performances by actively controlling vehicle motions. However, since vehicle sprung mass has six degrees of freedom (DOF) with strong couplings among them, it is hard to regulate individual motion state without affecting others [4–6]. While each chassis control system is designed for specific motion control and performance improvement, they may negatively impact others with potential conflict. In ABS design, for example, trade-off has to be made between stability and braking distance [7]. Cooperation and integration of the individual chassis subsystem have to be considered for further development of vehicle safety research [8].

There have been plenty of attempts to integrate the stand-alone chassis control subsystems, to name a few, the integrated chassis control (ICC) [9], unified chassis control (UCC) [10], vehicle dynamics management (VDM) [11], and so on. He et al. proposed a strategy to integrate active steering and Variable Torque Distribution (VTD) systems using the phase plane method, and a rule based integration scheme is employed to determine and allocate the control tasks between these two subsystems [12]. Wang et al. brought out an integrated control technology of vehicle chassis based on multiagent system (MAS) for coordination control between semiactive suspension (SAS) and electric power steering (EPS) [13].

Both yaw stability control and active front steering control of a vehicle play important roles in its stability. However, they have their own drawbacks as well. Thus, there were a lot of research works focusing on the cooperation between AFS and ESC controls to maintain vehicle desired yaw rate and side slip angle in order to improve vehicle handling and stability [14–19]. Cho et al. described a UCC system that consists of a supervisor and a coordinator to integrate AFS and ESC. The supervisor determines the target yaw rate and lateral velocity based on typical control modes, and the Karush-Kuhn-Tucker (KKT) condition is used to compute the optimized coordination of tire forces, considering constraint corresponding to the tire friction circle [20]. Ding and Taheri designed an adaptive integrated algorithm by integrating the AFS and DYC controls based on direct Lyapunov method [21]. Li and Yu designed a supervisory and servo-loop structure for the integration control of AFS and DYC [22]. The approach was used to reduce the conflicting effects of the two dynamically coupled subsystems.Doumiati et al. investigated the coordination of active front steering and rear braking in a driver-assist system for vehicle yaw control. The coordination of these actuators was achieved through a suitable gain scheduled LPV (Linear Parameter Varying) controller [23].

However, most of prior research has adopted a supervisory control method to coordinate the control commands and actuations in order to avoid control conflicts and to maximize resource sharing. In such cases, AFS and YSC control techniques are optimized individually in specific handling regions, and the maximum benefit could be gained through the coordinated/integrated use of both methods of corrective yaw motion generation in the control strategy. While this approach may be effective to some degree in reducing the interferences among multiple controlled subsystems and easing the conflicts among different control objectives, it is not a true “integration” per se; besides it has added an extra layer of command hierarchy on top of the stand-alone subsystems. The coupling mechanism and decoupling method for these two active control systems lack in-depth research. Actually, the integration of AFS and YSC, for example, is a typical two-input and two-output system with strong coupling in vehicle lateral dynamics; it is thus more desirable to decouple the dynamics so as to reduce or eliminate the control interference.

Furthermore, though easily and highly efficiently implemented, facing the nonlinear vehicle systems and the velocity and cornering stiffness variation, the traditional control system design methods lose their edge. However, as general solutions to the problems above, the modern control methods tend to have a complex control process. Besides, considering the heavy calculation burden, it is hard to achieve real-time control with some optimization control methods. Therefore, the improvement of classical control methods that fit the nonlinear requirement is what engineers have been researching [24, 25].

Inverse Nyquist Array (INA) method, a multivariable frequency method developed by Rosenbrock 1969 and further enhanced by Mac Farlance 1970, has been proved to be very effective in decoupling linear systems properly in both high and low frequency bands [26–28]. This method is of interest because it enables the utilization of classical single-loop systems for multivariable control system designs. After decoupling the plant by INA precompensator, the classical common control method for single-loop systems could be adopted. So, it has obtained widespread applications in the field of automatic control and industry [29–31].

However, the vehicle is a complex nonlinear system as is well known. As a linear-model-based control method, the controller designed by INA method shows less robust stabilities and cannot cover the complexity of vehicle states.

In this paper, an improved INA based feedback ICC controller is designed for AFS and YSC integration. First, a 2-DOF reference model is adopted. Based on this model, the plant of vehicle dynamics is decoupled by the precompensation INA method. The precompensator is solved with the consideration of variation of vehicle velocity and the cornering stiffness of both axles, which are functions of vehicle longitudinal and lateral accelerations of the target vehicle. Thus, the parameters in the linear 2-DOF reference model will change and the nonlinear characteristics of actual vehicle are taken into account. It means that the INA decoupling performance is regulated automatically based on different vehicle states as well as various system frequency bands. Then, the target yaw moment and the target front steering angle are achieved by the feedback PI controller. As analytic solutions of the precompensator are described explicitly, the execution efficiency of the ICC controller is pretty high. Finally, simulations are performed to validate the proposed method and the results are discussed.

## 2. Structure of INA Based Feedback Integrated Controller

**Figure 1:**
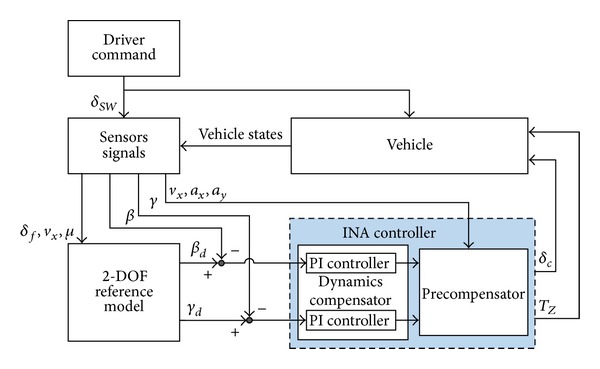
ICC system configurations.

The structure of the proposed ICC system for AFS and YSC integration is composed of a reference model and an INA based integrated controller which is shown in Figure 1, where *δ*_*SW*_ is steering wheel angle; *δ*_*f*_ is front wheel steer angle; *v*_*x*_,*v*_*y*_ are longitudinal and lateral velocity; *μ* is adhesion coefficient; *β* is sideslip angle; *γ* is yaw rate; *a*_*x*_, *a*_*y*_ are vehicle longitudinal, lateral acceleration; *δ*_*c*_ is active front wheel steering angle; *T*_*z*_ is the active yaw moment.

**Figure 2:**
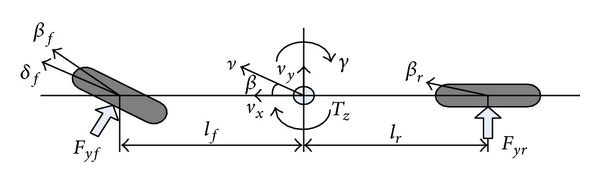
2-DOF reference model.

The 2-DOF reference vehicle model which considers both accuracy and simplicity is used for target inputs calculation. The side slip angle and yaw rate are described in the model, as shown in Figure 2.

The vehicle state space equation is

(1) $$\begin{array}{c} \dot{x}=Ax+Bu,\\ y=Cx, \end{array}$$

where

(2) $$\begin{array}{c} x={\left[\begin{array}{cc} \beta & \gamma \end{array}\right]}^{T},\\ u={\left[\begin{array}{cc} \delta_{f} & T_{Z} \end{array}\right]}^{T},\\ A=\left[\begin{array}{cc} a_{11} & a_{12}\\ a_{21} & a_{22} \end{array}\right]=\left[\begin{array}{cc} -\frac{c_{f}+c_{r}}{m\cdot v_{x}} & -1+\frac{c_{r}l_{r}-c_{f}l_{f}}{m\cdot v_{x}^{2}}\\ \frac{c_{r}l_{r}-c_{f}l_{f}}{I_{z}} & -\frac{c_{r}l_{r}^{2}+c_{f}l_{f}^{2}}{I_{z}\cdot v_{x}} \end{array}\right],\\ B=\left[\begin{array}{cc} b_{11} & b_{12}\\ b_{21} & b_{22} \end{array}\right]=\left[\begin{array}{cc} \frac{c_{f}}{m\cdot v_{x}} & 0\\ \frac{c_{f}l_{f}}{I_{z}} & \frac{1}{I_{z}} \end{array}\right],\\ C=\left[\begin{array}{cc} c_{11} & c_{12}\\ c_{21} & c_{22} \end{array}\right]=\left[\begin{array}{cc} 1 & 0\\ 0 & 1 \end{array}\right], \end{array}$$

where *c*_*f*_, *c*_*r*_ are front and rear axle cornering stiffness; *m* is mass; *l*_*f*_, *l*_*r*_ describe the distances from the vehicle c.g. (center of gravity) to the front and rear axle, respectively; *I*_*z*_ is yaw inertia of the vehicle; *β*_*f*_, *β*_*r*_ are front and rear slip angle; *F*_*yf*_,*F*_*yr*_ are lateral force of the front and rear axle.

To maintain lateral stability, both the yaw rate and side slip angle should be restricted within a stable field. The desired yaw rate can be obtained from steady-state yaw rate gain of the reference model:

(3) $$\begin{array}{c} \gamma_{d}=\frac{v_{x}\cdot \delta_{f}}{\left(l_{f}+l_{r}\right)\cdot \left[1+{\left(v_{x}/v_{ch}\right)}^{2}\right]}, \end{array}$$

where

(4) $$\begin{array}{c} v_{ch}^{2}=\frac{c_{f}\cdot c_{r}\cdot {\left(l_{f}+l_{r}\right)}^{2}}{m\left(c_{r}l_{r}-c_{f}l_{f}\right)}. \end{array}$$

In addition, the desired yaw rate should be constrained by the road friction coefficient:

(5) $$\begin{array}{c} \left|\gamma_{d}\right|\leq \mu \cdot \frac{g}{v_{x}}. \end{array}$$

To maintain lateral stability, it is important to sustain driver's control authority, which can be achieved when the vehicle sideslip angle is small. According to some literatures [15, 32], the desired sideslip angle can be chosen as

(6) $$\begin{array}{c} \beta_{d}=0. \end{array}$$

Furthermore, the same 2-DOF model is also used for the INA controller design. By Laplace transformation, the state equation (1) can be rewritten into the system Transfer Function Matrix (TFM) as

(7) G(s) =C(sI−A)−1B+D =[g11(s)g12(s)g21(s)g22(s)] =[(b11s−b11a22+b21a12)(s−a11)(s−a22)−a21a12b22a12(s−a11)(s−a22)−a21a12(b21s+b11a21−b21a11)(s−a11)(s−a22)−a21a12b22(s−a11)(s−a11)(s−a22)−a21a12]

**Figure 3:**
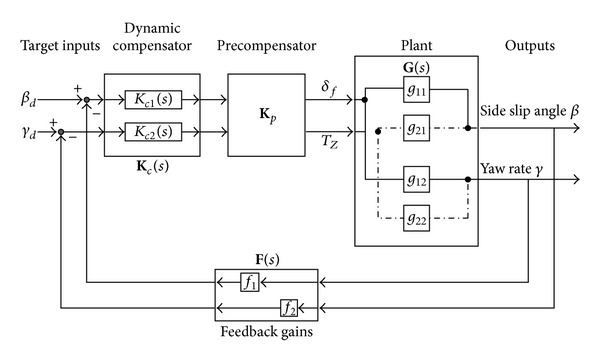
Structure of INA controller.

Obviously, it is a typical two-input, two-output system, and **G**(*s*) can be decoupled or pseudodecoupled in overall frequency bands by INA method. The structure of the INA controller designed in this paper is shown in Figure 3. An appropriate precompensator **K**_*p*_ is designed to make the system TFM diagonal dominance, that is, to decouple the control plant. Then the design methods for classical SISO systems can be used consequently [28]. In this research, a dynamic compensator matrix **K**_*c*_ and a feedback gain matrix **F** for the decoupled MIMO system are designed utilizing the classical SISO feedback PI control methods.

In Figure 3,

(8) $$\begin{array}{c} K_{c}\left(s\right)=\left[\begin{array}{cc} K_{c1}\left(s\right) & 0\\ 0 & K_{c2}\left(s\right) \end{array}\right],\\ F\left(s\right)=\left[\begin{array}{cc} f_{1}\left(s\right) & 0\\ 0 & f_{2}\left(s\right) \end{array}\right]. \end{array}$$

In this work, unity feedbacks should be adopted between reference inputs and actual inputs; that is,

(9) $$\begin{array}{c} F\left(s\right)=I_{2}. \end{array}$$

## 3. Design of Nonlinear Modified INA System

**Figure 4:**
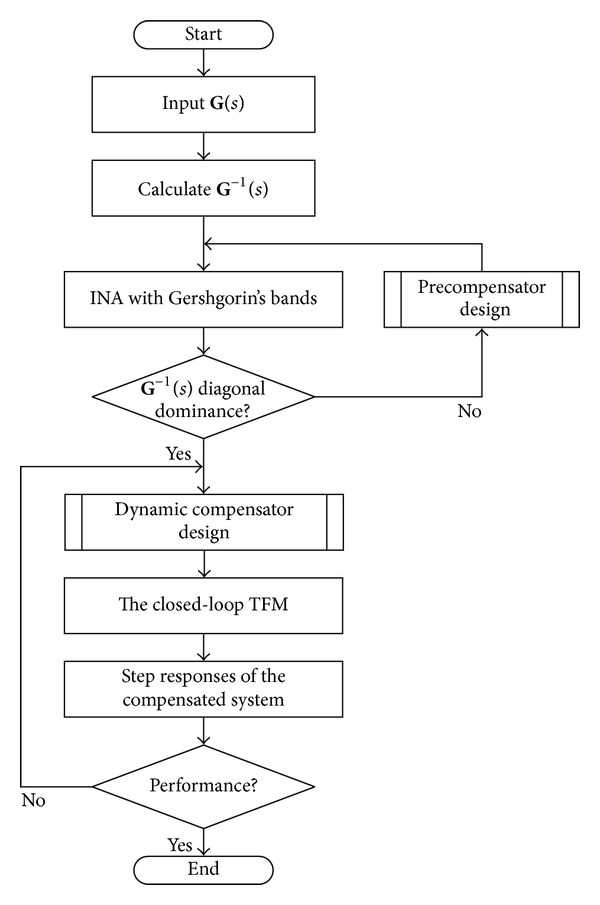
INA controller design flow.

The INA controller design flow is demonstrated in Figure 4. First, the appropriate precompensator **K**_*p*_ is designed to decouple the vehicle system** G**(*s*). Being compensated, the inverse system transfer function **K**_*p*_^−1^(*s*) **G** ^−1^(*s*) should be diagonal dominance, which can be judged by Gershgorin's bands and Diagonal Dominance Factors. Then, the PI dynamic compensator matrix **K**_*c*_ is designed. The parameters of the PI controller should be set by the analysis of bode graphs of open-loop system and the step responses of the decoupled close-loop system.

A square matrix **Q**(*s*) is said to be of diagonal dominance on a contour *D* if, for each column (or row), the modulus of the diagonal element is larger than the sum of the modulus of the off-diagonal elements for each complex variable *s* in *D*:

(10) |qii(s)|>∑j=i(j≠i)m|qij(s)| (i=1,2,…,m).

According to Gershgorin's theorem [27], for specific *s*, Gershgorin's circles corresponding to *m* column (or row) of **Q**(*s*) have the centres of *q*_*ii*_(*s*) and the radii of *r*_*i*_(*s*):

(11) $$\begin{array}{c} r_{i}\left(s\right)=\overset{m}{\underset{\begin{array}{c} j=1\\ \left(j\neq i\right) \end{array}}{\sum }}\left|q_{ij}\left(s\right)\right|\;\left(i=1,2,\ldots ,m\right). \end{array}$$

As *s* runs through the contour *D*, the corresponding Gershgorin's circles for the *m* columns (or rows) will sweep out *m* bands centered by the trajectories *q*_*ii*_(*s*), respectively. These bands are called Gershgorin's bands of the matrix **Q**(*s*). It is clear that Gershgorin's bands will not include the origin of the complex *s*-plane and **Q**(*s*) will be nonsingular for any value of *s* on a contour *D* if **Q**(*s*) is diagonal dominance.

Gershgorin's bands of the inverse TFM **G** ^−1^(*s*) can be achieved by selecting Nyquist *D*-contour.

**Figure 5:**
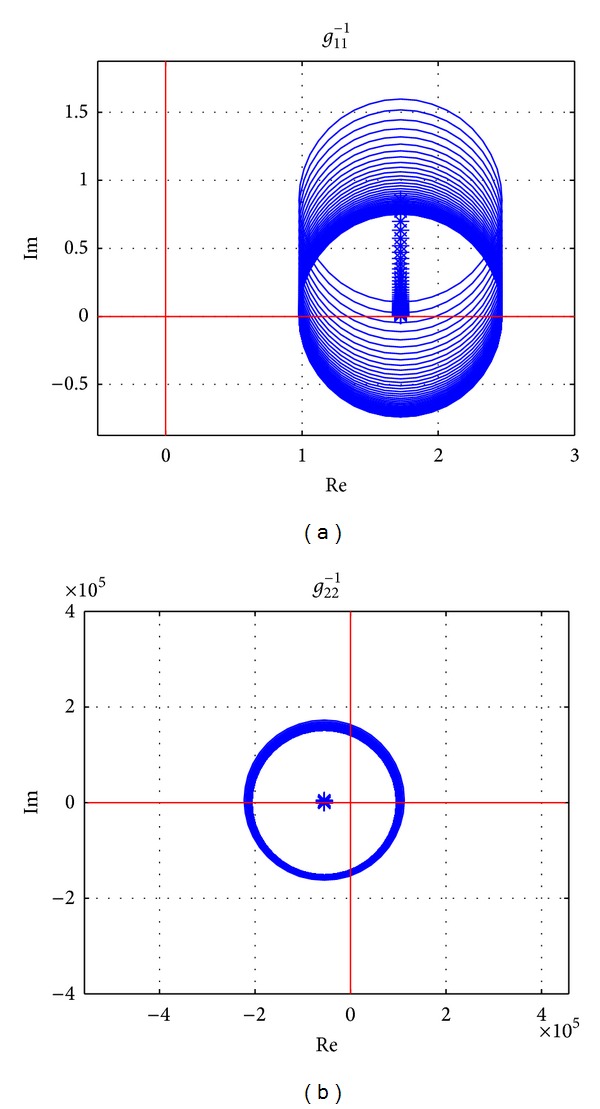
Uncompensated INA with Gershgorin's bands, at *v*_*x*_ = 100 km/h, frequency from 0.01 Hz to 10 Hz.

Using the parameters in Table 1, Gershgorin's bands of **G** ^−1^(*s*) when the vehicle is driving at longitudinal speed 100 km/h are drawn in Figure 5. It is seen that the original point of the complex plane is not included in Gershgorin's bands of *g*_11_^−1^(*s*) which is corresponding to the input of *T*_*z*_. It means there is weak coupling for the vehicle system from the direct yaw moment input on the sprung mass. However, for *g*_22_^−1^(*s*), which is corresponding to the input of front steering wheel angle *δ*_*f*_, the origin of *s*-plane is located in Gershgorin's bands, and strong coupling will be shown. It is concluded that the steering input affects both yaw rate and side slip angle while the direct yaw moment affects yaw rate only, which can also be dedicated from (1) clearly.

In order to analyze the diagonal dominance of the plant, the Diagonal Dominance Factor (DDF) is introduced. The DDF is defined as

(12) $$\begin{array}{c} f_{dd}=\frac{\left|g_{ii}\left(s\right)\right|}{\sum_{j=1}^{m}\left|g_{ij}\left(s\right)\right|}\;\left(i=1,2,\ldots ,m\right). \end{array}$$

**Figure 6:**
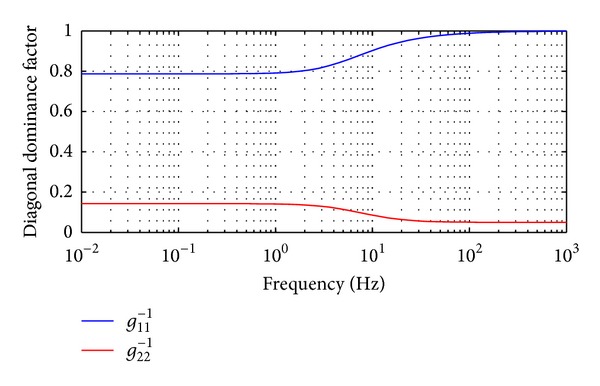
Diagonal Dominance Factors for uncompensated INA, *v*_*x*_ = 100 km/h.

The DDF of uncompensated **G** ^−1^(*s*) is shown in Figure 6. It is clear that the diagonal dominance of 1st row of **G** ^−1^(*s*) is weaker at lower frequency and is stronger at higher frequency, while it is the opposite for the 2nd row. In order to achieve perfect decoupling through overall frequency bands, the precompensations should be applied from lower to higher frequency fields. In this paper, the precompensator is designed according to the decoupling analysis of TFM at frequency point 0 and +*∞*.

At frequency point 0, the precompensator matrix can be achieved easily by

(13) $$\begin{array}{c} K_{l}=G^{-1}\left(0\right). \end{array}$$

However, when the frequency is near to +∞, the system cannot be decoupled directly by the method of (13) as

(14) $$\begin{array}{c} G\left(\infty \right)=\underset{s\to \infty }{\lim}G\left(s\right)=0 \end{array}$$

which means that **G**(*∞*) is irreversible. Thus, a different method is necessary.

Rewrite matrix **G** ^−1^(*s*) as

(15) $$\begin{array}{c} G^{-1}\left(s\right)=\frac{1}{d\left(s\right)}P\left(s\right)=\frac{1}{d\left(s\right)}\left[\begin{array}{cccc} p_{1}\left(s\right) & p_{2}\left(s\right) & \cdots & p_{m}\left(s\right) \end{array}\right], \end{array}$$

where **P**(*s*) is a polynomial matrix, *p*_*i*_(*s*) are column vectors of **P**(*s*), and *d*(*s*) is the least common denominator of elements of **G** ^−1^(*s*).

Let *r*_*i*_ be the highest degrees of the elements of each *p*_*i*_(*s*); the precompensator matrix for high frequency is

(16) $$\begin{array}{c} K_{h}=\left[\begin{array}{cccc} \frac{p_{1}\left(s\right)}{s^{r_{1}}} & \frac{p_{2}\left(s\right)}{s^{r_{2}}} & \cdots & \frac{p_{m}\left(s\right)}{s^{r_{m}}} \end{array}\right],\;\text{as }s⟶\infty . \end{array}$$

Thus, by integrating **K**_*l*_ and **K**_*h*_, the system can be compensated perfectly from frequency 0 to +*∞*. The precompensator is

(17) $$\begin{array}{c} K_{p}=\frac{1}{s}K_{l}+K_{h}. \end{array}$$

The compensated INA becomes

(18) $$\begin{array}{c} Q^{-1}\left(s\right)=K_{p}^{-1}\left(s\right)\cdot G^{-1}\left(s\right). \end{array}$$

**Figure 7:**
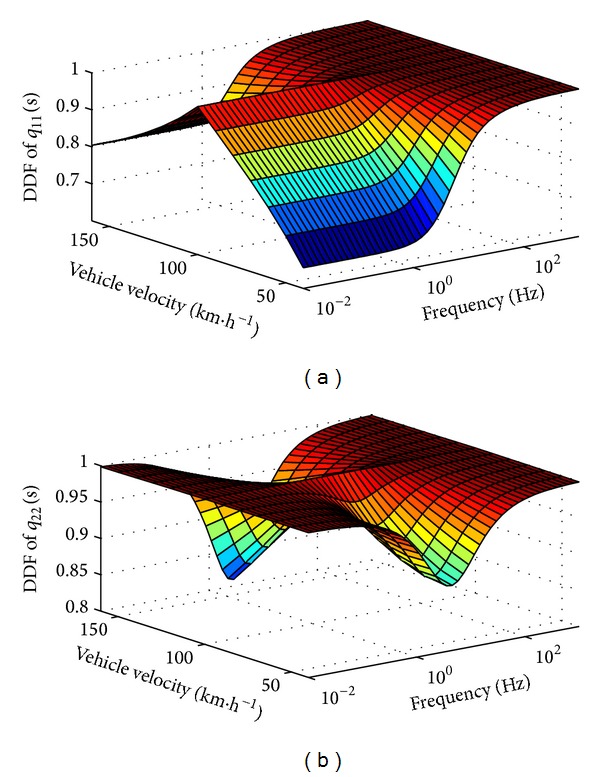
DDF for *q*_11_(*s*) and *q*_22_(*s*) of **Q** ^−1^(*s*), without the consideration of nonlinear characteristics.

By designing the precompensator from (13) to (17) with specific *v*_*x*_ = 100 km/h, the DDF of compensated **Q** ^−1^(*s*) is shown in Figure 7.

It is seen that both of the DDFs of *q*_11_(*s*) and *q*_22_(*s*) are almost 1 when the *v*_*x*_ in the plant is 100 km/h, which means that the system is decoupled perfectly at this velocity. However, the DDFs decrease as *v*_*x*_ departing from 100 km/h, especially in lower and middle frequency fields. As shown in Figure 7(a), the DDF of *q*_11_(*s*) decreased to 0.66 at frequency = 0.01 Hz and *v*_*x*_ = 40 km/h, while in Figure 7(b), the DDF of *q*_22_(*s*) decreased to 0.81 at about frequency = 2.81 Hz and *v*_*x*_ = 160 km/h. It is clear that the performance of the compensator was affected negatively by the nonlinear character caused by the variation of vehicle velocity.

Using (7) and (13) to (17), the precompensator can be solved analytically. By substituting (7) to (13) and (15)-(16), respectively, we get

(19) $$\begin{array}{c} K_{l}=\left[\begin{array}{cc} \frac{c_{f}+c_{r}}{c_{f}} & \frac{mv_{x}^{2}+c_{f}a+c_{r}b}{v_{x}c_{f}}\\ -c_{r}\left(a+b\right) & \frac{-\left(amv_{x}^{2}-c_{r}b^{2}+c_{r}ab\right)}{v_{x}} \end{array}\right],\\ K_{h}=\left[\begin{array}{cc} \frac{v_{x}m}{c_{f}} & 0\\ -v_{x}am & I_{z} \end{array}\right]. \end{array}$$

*v*_*x*_ can be achieved from the actual vehicle or vehicle model directly, while the cornering stiffness of each axle also changes on vehicle driving.

**Figure 8:**
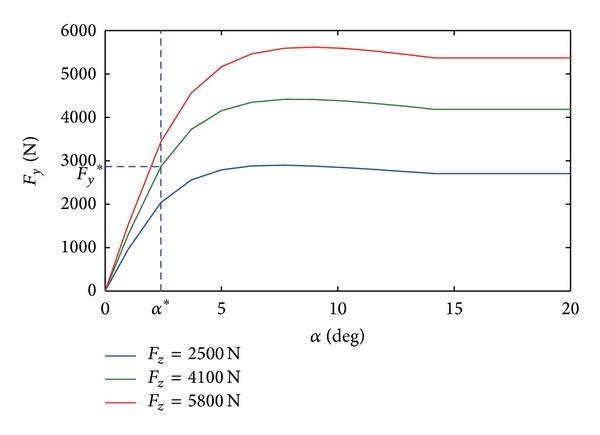
Curves for tire cornering characteristic.

As is well known, the tire cornering force can be described as map of tire vertical load, road friction coefficient, and tire slip angle:

(20) $$\begin{array}{c} c=\frac{F_{y}^{*}}{\alpha^{*}}, \end{array}$$

where *F*_y_* and α* are coordinate values of specific point on linear section of tire cornering characteristic curve at given vertical load *F*_*z*_, which is shown in Figure 8.

The vertical load of each wheel is calculated by the following equations, and then the cornering force of each wheel can be interpolated according to data chart in Figure 8:

(21) Fzfl=mg·lr2(lf+lr)−max·hg2(lf+lr) −kfΦkfΦ+krΦ·(mayhg+mghrΦd),Fzfr=mg·lr2(lf+lr)−max·hg2(lf+lr) +kfΦkfΦ+krΦ·(mayhg+mghrΦd),Fzrl=mg·lr2(lf+lr)+max·hg2(lf+lr) −kfΦkfΦ+krΦ·(mayhg+mghrΦd),Fzrr=mg·lr2(lf+lr)+max·hg2(lf+lr) +kfΦkfΦ+krΦ·(mayhg+mghrΦd),

where *F*_*zfl*_, *F*_*zfr*_, *F*_*zrl*_, and *F*_*zrr*_ are vertical load on front left, front right, rear left, and rear right tire, respectively; *d* is wheel track; *h*_*g*_ is height of center of sprung mass; *h*_*r*_ is the distance between height of center of sprung mass and roll center; *k*_*f*Φ_ and *k*_*r*Φ_ are roll stiffness of front and rear axle, respectively; Φ is roll angle.

Then

(22) $$\begin{array}{c} c_{f}=\frac{F_{yfl}^{*}+F_{yfr}^{*}}{\alpha^{*}}=f\left(F_{zfl}^{*}+F_{zfr}^{*}\right),\\ c_{r}=\frac{F_{yrl}^{*}+F_{yrr}^{*}}{\alpha^{*}}=f\left(F_{zrl}^{*}+F_{zrr}^{*}\right), \end{array}$$

where *f*(·) is the mapping function according to data chart Figure 8. Thus, the cornering stiffness is depicted as functions of vehicle longitudinal and lateral accelerations.

**Figure 9:**
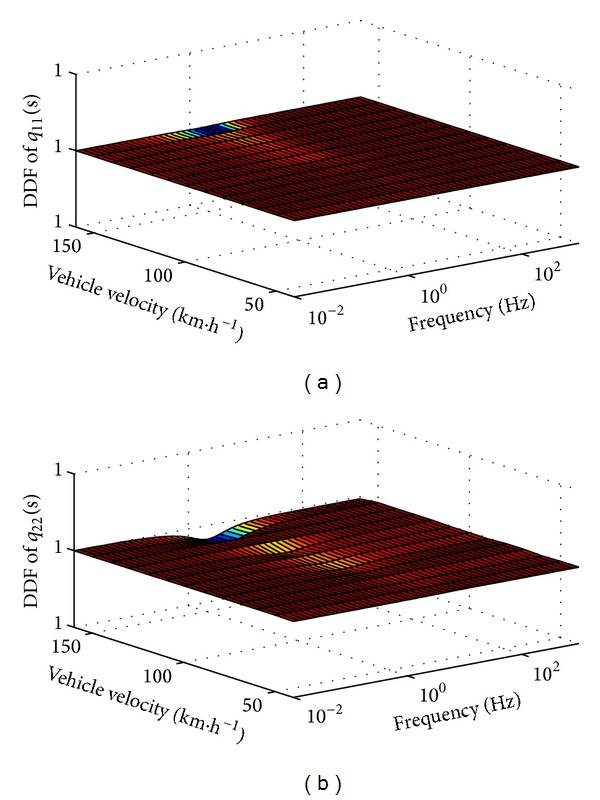
DDF for *q*_11_(*s*) and *q*_22_(*s*) of **Q** ^−1^(*s*), with the consideration of nonlinear characteristics.

With the consideration of nonlinear characteristics, the DDF of precompensated **Q** ^−1^(*s*) is shown in Figure 9. The DDFs of both diagonal elements are so close to 1 that the tiny deviation cannot be displayed in the ticks of vertical axis in the figures. In fact, the system is decoupled completely by the analytical solution and the deviation is caused by computer precision. It means that the outputs of vehicle yaw rate and side slip from both control inputs are almost completely decoupled in common vehicle speed region.

**Figure 10:**
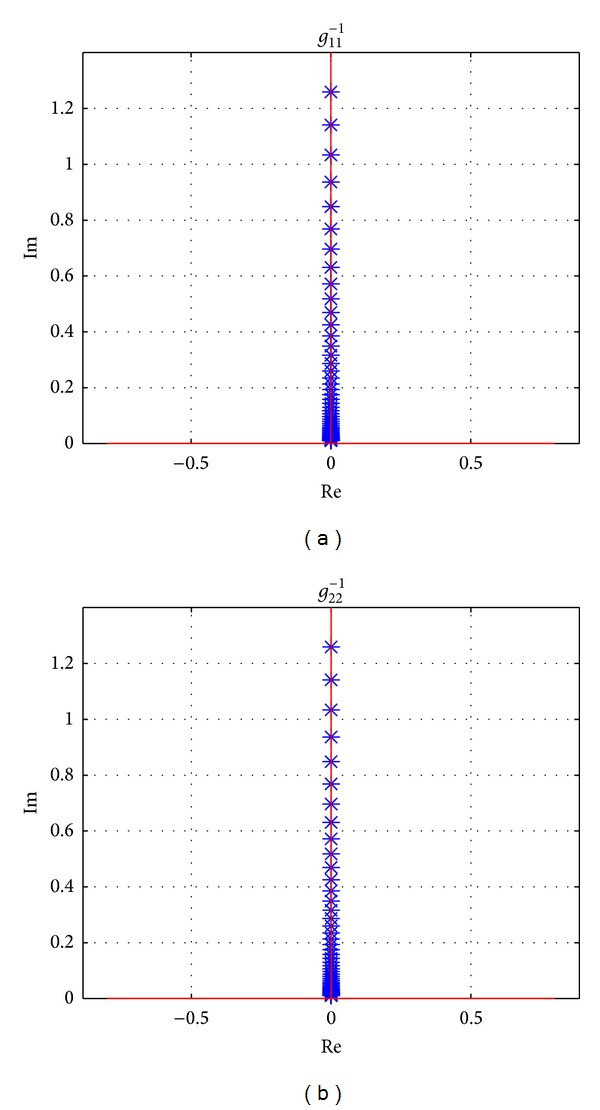
Gershgorin's bands for **Q** ^−1^(*s*), at *v*_*x*_ = 40 km/h, frequency from 0.01 Hz to 10 Hz.

**Figure 11:**
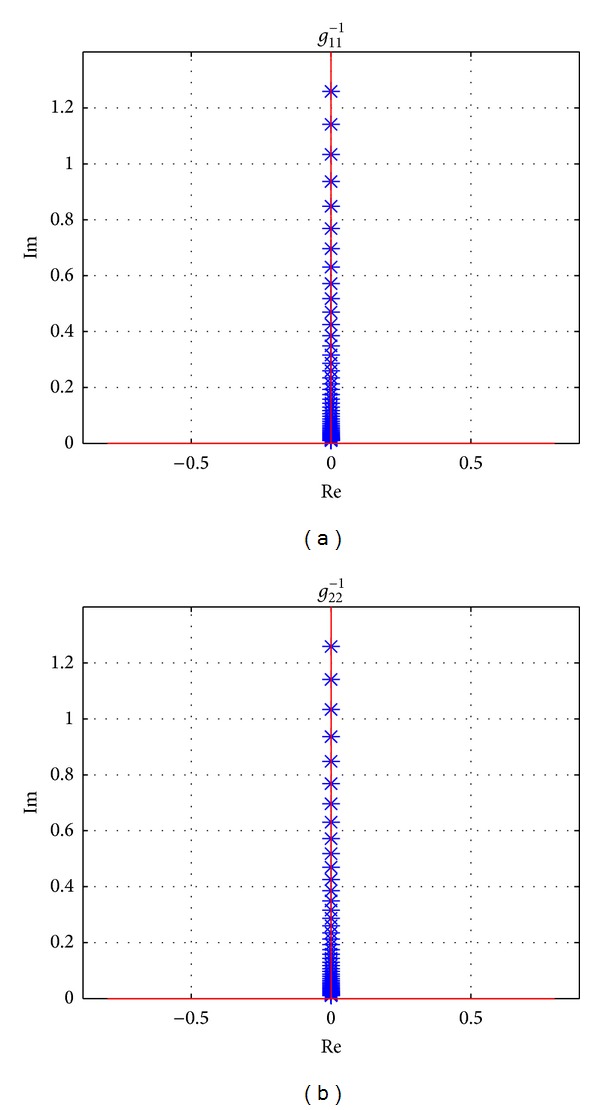
Gershgorin's bands for **Q** ^−1^(*s*), at *v*_*x*_ = 100 km/h, frequency from 0.01 Hz to 10 Hz.

**Figure 12:**
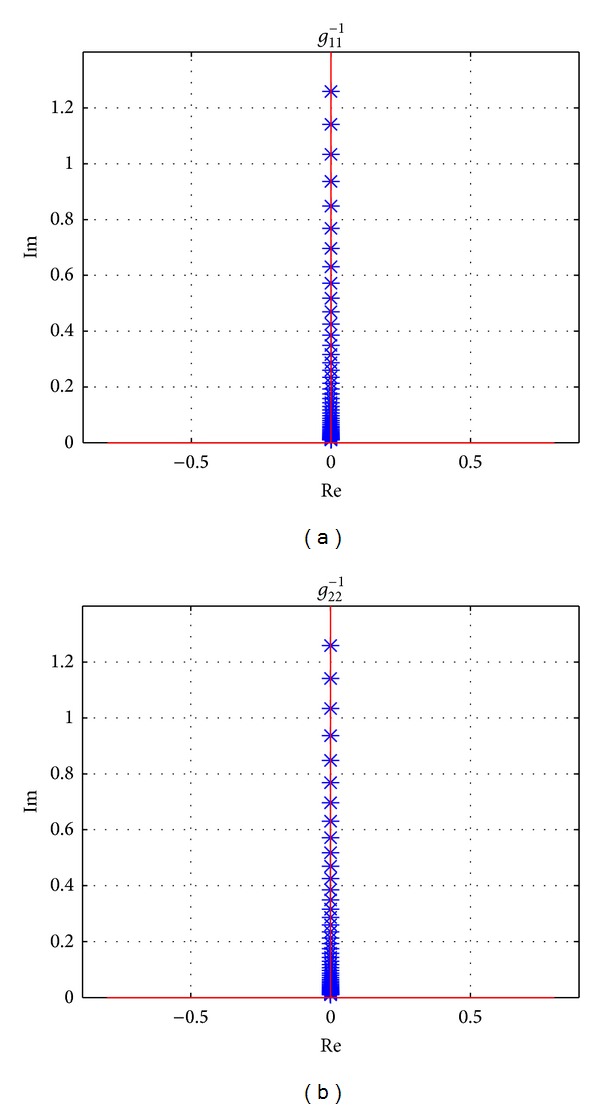
Gershgorin's bands for **Q** ^−1^(*s*), at *v*_*x*_ = 160 km/h, frequency from 0.01 Hz to 10 Hz.

**Figure 13:**
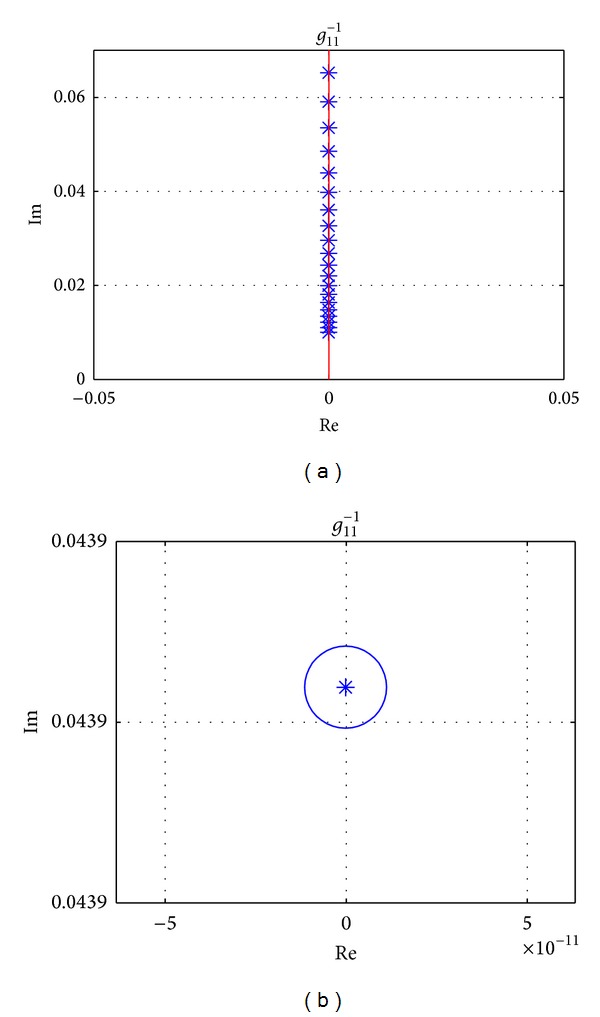
Detailed section of *g*_11_^−1^ in Figure 12.

The effectiveness of proposed precompensator is also proved by Gershgorin's bands at speeds 40 km/h, 100 km/h, and 160 km/h, which are shown in Figures 10, 11, and 12, respectively. And the detailed sections of Figure 12 are shown in Figure 13. All of Gershgorin's circles are tiny and keep origin of* s*-plane outside; thus, the system is perfect diagonal dominance.

As the system has been decoupled completely, the feedback PI controller design method for SISO is used to compensate the system dynamics characteristic. The TFM of the PI controller is

(23) $$\begin{array}{c} K_{c}\left(s\right)=K_{cP}+K_{cI}\frac{1}{s}=\left[\begin{array}{cc} k_{cp1} & 0\\ 0 & k_{cp2} \end{array}\right]+\left[\begin{array}{cc} k_{ci1} & 0\\ 0 & k_{ci2} \end{array}\right]\frac{1}{s}. \end{array}$$

Thus TFM for the INA based integrated controller is

(24) U(s)Δ(s)=KINA=Kc(s)·KP(s)=(Kh+Kl1s)(KcP+KcI1s)=KhKcPs2+(KlKcP+KhKcI)s+KlKcIs2.

For the convenience of programming, rewrite the controller TFM into state function mode:

(25) $$\begin{array}{c} {\dot{x}}_{\xi }=A_{\xi }x_{\xi }+B_{\xi }\Delta ,\\ u=C_{\xi }x_{\xi }+D_{\xi }\Delta , \end{array}$$

where

(26) $$\begin{array}{c} x_{\xi }={\left(\begin{array}{cccc} x_{\xi 1} & x_{\xi 2} & x_{\xi 3} & x_{\xi 4} \end{array}\right)}^{T}\\ \Delta =x_{d}-x,\;\;x_{d}={\left(\begin{array}{cc} \delta_{d} & \gamma_{d} \end{array}\right)}^{T},\\ A_{\xi }=\left[\begin{array}{cc} 0 & 0\\ K_{cI} & 0 \end{array}\right],\;\;B_{\xi }=\left[\begin{array}{c} I_{2}\\ K_{cP} \end{array}\right],\\ C_{\xi }=\left[\begin{array}{cc} K_{h}K_{cI} & K_{l} \end{array}\right],\;\;D_{\xi }=K_{h}K_{cP}. \end{array}$$

**x** and **u** were defined in (1).

## 4. Analysis and Simulation

**Figure 14:**
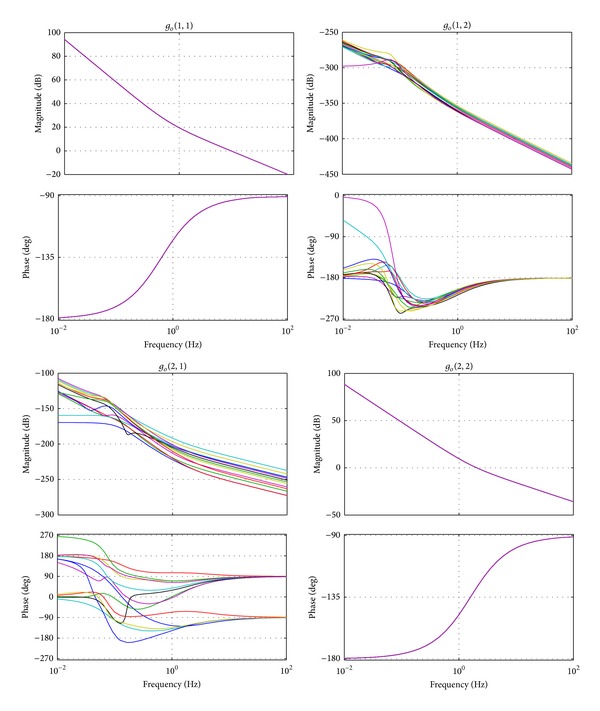
Bode graphs of the open-loop system.

Applying the proposed controller, which is described by (25), to the 2-DOF system, which is described by (1), the bode graphs of the open-loop system *g*_*o*_(*s*) with different *v*_*x*_ = 40, 50, …, 160 are shown in Figure 14. The open-loop response of *g*_*o*_(1,1) and *g*_*o*_(2,2) can be analyzed from the top left and bottom right graphs, respectively.

It is seen that gains *g*_*o*_(1,2) and *g*_*o*_(2,1) are close to zero, which implies that the system has been decoupled completely, and only the responses of *g*_*o*_(1,1) and *g*_*o*_(2,2) should be considered for the performance of the controlled system. The same conclusion can be drawn from the fact that the gain lines and phase lines of different *v*_*x*_ are almost coincident and are not affected by the minor diagonal elements of system TFM.

**Figure 15:**
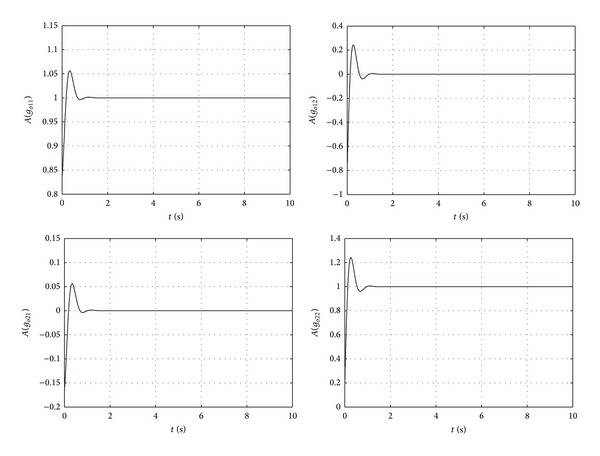
Step responses of the close-loop system.

The unit step response of the close-loop system *g*_*c*_(*s*) is shown in Figure 15. It is seen that the responses of *g*_*c*_(1,1) and *g*_*c*_(2,2) converge to 1 and the responses of *g*_*c*_(1,2) and *g*_*c*_(2,1) converge to 0 quickly. By setting the PI controller **K**_**c**_(*s*) carefully, the time domain response of the close-loop system can be regulated to a satisfied level.

**Figure 16:**
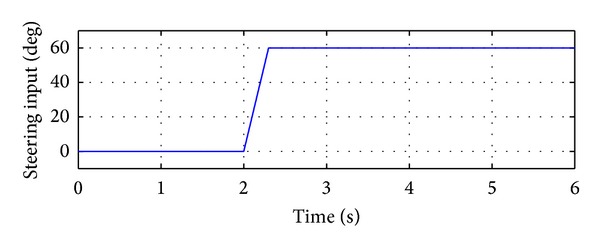
Steering wheel inputs for step steering simulation.

Simulations were carried out by Matlab/Simulink. A 2-DOF vehicle model and a 14-DOF vehicle model were programmed and the step steering maneuver is simulated by both vehicle models, respectively [33–35]. At 2 s, a step steering input with amplitude of 60 deg. was applied with 0.3 s. The steering wheel input is shown in Figure 16. Both simulations are performed on a road with a friction coefficient of 0.5. The simulations lasted to 6 s.

**Figure 17:**
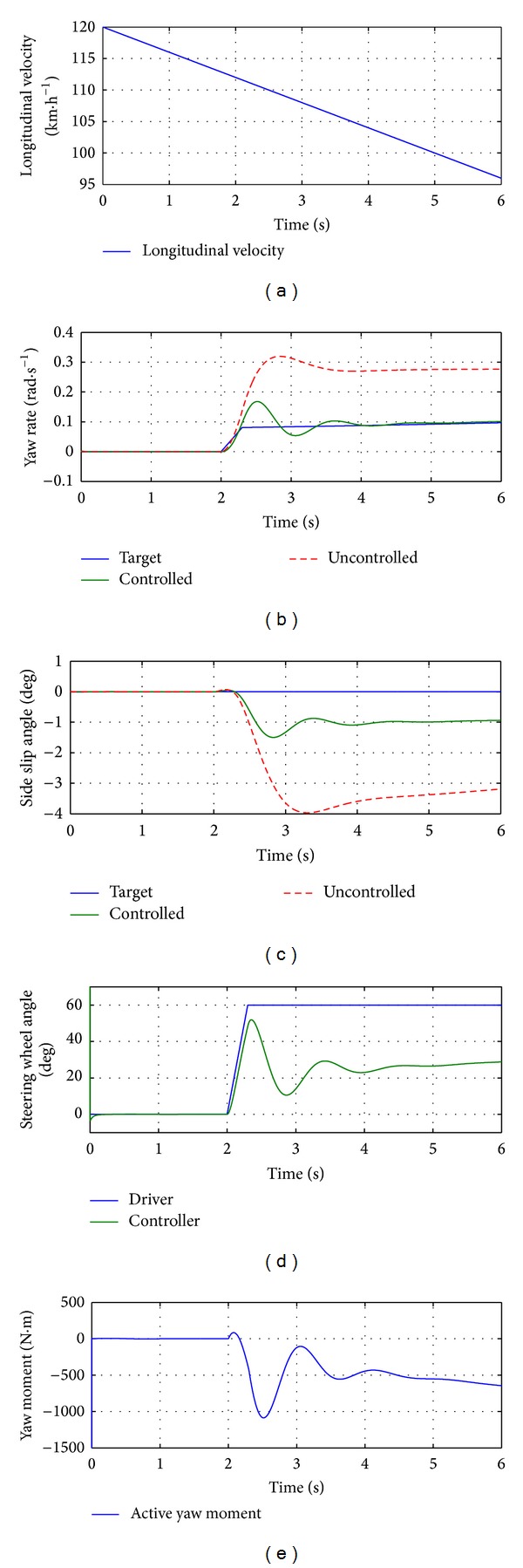
Simulation results for 2-DOF vehicle model.

The simulation results of 2-DOF model are shown in Figure 17. During the simulation, the longitudinal velocity of the vehicle was set as decreasing linearly at the rate of 4 km/h from 120 km/h, which is shown in Figure 17(a). The yaw rate and side slip angle of target value, controlled and uncontrolled values are shown in Figures 17(b) and 17(c). Figures 17(d) and 17(e) show the steering wheel and active yaw moment from the controller. It is seen that the steering wheel and active yaw moment control are integrated by the proposed controller, and both yaw rate and side slip angle of the vehicle are reduced and closer to target values.

**Figure 18:**
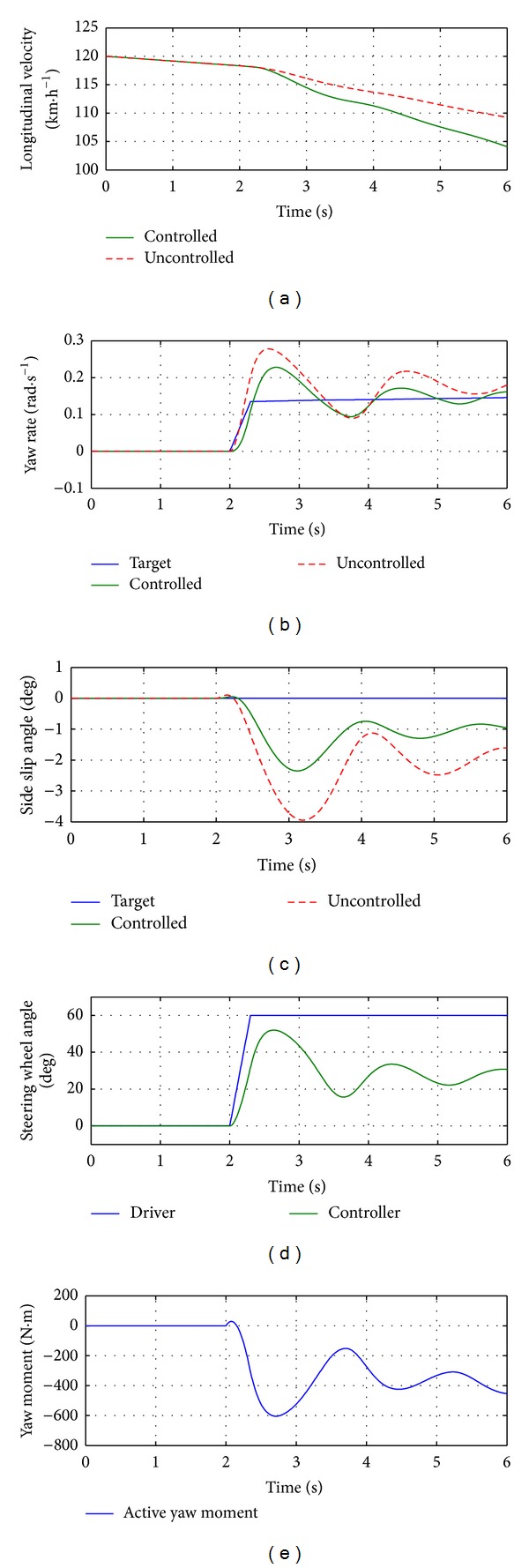
Simulation results for 14-DOF vehicle model.

While simulating with 14-DOF model, the longitudinal velocity of the vehicle is generated automatically, which is shown in Figure 18(a). From Figures 18(b) to 18(e), the similar behaviors of the controller and vehicle in spite of larger fluctuations are shown. The ICC coordinated the AFS and YSC system effectively and the side slip angle and yaw rate were regulated to a desired region.

## 5. Conclusion

This paper presents a control algorithm for integration of AFS and YSC systems based on a 2-DOF vehicle model. The coupling characteristic of the typical two-input, two-output system is analyzed, and it is decoupled and compensated by the INA design method. In the research, we found that although the traditional INA controller could decouple the system well in given circumstances, the performance of the compensator was affected negatively by some kinds of nonlinear factors. Therefore, the nonlinear characteristics caused by the change of velocity and cornering stiffness were considered which implies that the improved INA method can be qualified for the nonlinear vehicle driving maneuvers. The correction of the precompensator ensures the effectiveness of the controller covering overall frequency bands and vehicle speeds. As the analytic format of the precompensator is proposed, the decoupling algorithm runs fast.

After being decoupled, a PI feedback controller is designed using the traditional design method for SISO linear systems. The frequency response of the open-loop system and the step response on time domain of the close-loop system were analyzed. The results show that the stability and response characteristics of the system are guaranteed by the feedback gain matrix and dynamic compensation matrix.

Finally, simulations were carried out using a 2-DOF model and a 14-DOF model by Matlab/Simulink. The simulation results of step steering indicate that the proposed ICC control can reduce yaw and side slip of the target vehicle significantly and improve its handling and stability consequently.

## Figures and Tables

**Table 1:** Vehicle parameters for Gershgorin's bands calculation.

Parameters	Symbol	Values
Mass (kg)	*m*	1530
Front axle cornering stiffness (N/rad)	*c*_*f*_	75435
Rear axle cornering stiffness (N/rad)	*c*_*r*_	54594
Distance from vehicle CG to the front axle (m)	*l*_*f*_	1.11
Distance from vehicle CG to the rear axle (m)	*l*_*r*_	1.67
Vehicle moment of inertia about the *z*-axis (kgm^2^)	*I*_*z*_	4192
